# Supplementary material for: N2 sleep promotes the occurrence of ‘aha’ moments in a perceptual insight task
Source: PLoS Biol. 2025 Jun 26;23(6):e3003185. doi: 10.1371/journal.pbio.3003185 (PMC12200826; doi:10.1371/journal.pbio.3003185)
Supplement: Attachment — Submitted filename: Loewe_etal_Revision_PointbyPoint.pdf [file pbio.3003185.s002.pdf]

We would like to thank you and the reviewers for your thorough reading, helpful comments and suggestions. We respond to all issues in our point-by-point reply, where we first reproduce the reviewers' comments and followed our corresponding responses to each of them. Changes to the manuscript are also reproduced next to the original text below and highlighted in yellow in the accompanying manuscript file.

Reviewer 1

Comment 1: In addition to sleep stage, may also the kind of task/task structure matter in how sleep contributes to insight? Could it be that task where renormalization or a relaxation of constraints is key benefit most strongly from sleep? Could task where a statistical regularity needs to be discovered benefit most strongly from sleep? Since the authors briefly touch on this already in the discussion (comparing their task and the NRT findings by Lacaux et al.) it might be worth introducing this additional idea already in the introduction. It may also explain why some studies did not find effects of sleep on insight (all using different tasks/problems).

Response:
We thank the reviewer for raising this important point. We agree that different tasks differ in the exact process(es) that underlie participants' insights, and that this has implications for the interpretation of our findings. We now mention a potential task dependence of sleep interventions on insight in the introduction on page 2:

Changed from…	Changed to…
	"Another possibility is that different cognitive tasks benefit differently from sleep. Lerner & Gluck (2019), for instance, have argued that sleep is particularly important for extracting hidden task regularities, in line with work that has found the strongest sleep effects in tasks that require extracting statistical rules. This would suggest that insight tasks, which mainly rely on learning implicit associative rules (such as the PSSST we tested in this study), benefit more from sleep."

We further elaborate on potential task differences in the Discussion on page 12:

Changed from…	Changed to…
	"Beyond sleep stages, different cognitive tasks might benefit from sleep in different ways – and potentially explain divergent findings regarding the relationship of sleep and insight. For instance, sleep has been found to aid in

	statistical learning (Durrant, Taylor, Cairney, & Lewis, 2011) and particularly in the extraction of hidden task regularities (Lerner & Gluck, 2019).<br>Besides the associative learning of hidden structure in the PSSST and NRT, participants also need to overcome the initially learned strategy, which could block learning of the associations required for the insight (Kamin, 1969; Mackintosh, 1973). Both downscaling of redundant information in terms of weak synaptic connections, as well as global downscaling of all synaptic weights to a speculative ‘clean slate' could potentially account for the post sleep insight. In favour of the clean slate hypothesis would be that subjects often take a while after waking up to discover the hidden rule (Wagner et al. 2004, Lacaux et al., 2021), i.e. they don't wake up with the insight solution.”

Comment 2: What were the differences in paradigm between previous study and sleep study? Was there a delay time? If not, this comparison must be treated with caution because incubation in wake might similarly facilitate insight. I concur with the authors (although the group allocation was not randomized) that a comparison of participants who slept with those who did not fall asleep, in combination with their previous results, strongly suggests that sleep was key here. Please add a note of caution to the discussion/results section that this was not an experimental manipulation and strictly, causality can thus not be inferred. The correlative evidence, however, is strong and highly convincing.

Response:
We thank the reviewer for raising this important point.
There was no delay time in the previous study (Löwe et al., 2024), which we have now made more clear in the Results on page 4. The two experiments merely differed in the employed motion coherence levels (5/23/41/59/76 in Loewe et al, 2024, instead of 5/10/20/30/45), and the onset of the colour correlation which happened half a block earlier in the sleep study. Concerning the latter point we note that the difference is fairly subtle (550 trials of exposure to correlation in Loewe et al., 2024 vs. 600 trials here), that the frequency observed in the wake group generally concurs across the two experiments (49% in Loewe et al., 2024 vs 55% in the present study), and that a comparison of insight frequency between the N2 group and the Wake group was significant by itself.

Changed from…	Changed to…
“Notably, this percentage is substantially higher than a baseline of 49.5\% (49/99) insight that we observed in our previous study with closely related experimental procedures, but without a nap period [...].”	“Notably, this percentage is substantially higher than a baseline of 49.5\% (49/99) insight that we observed in our previous study with closely related experimental procedures, but without any nap or other delay period [...].”

It is true that incubation might facilitate insight, and a randomized assignment would have been ideal. However we did not see any beneficial effects of wake rest on insight in participants who did not fall asleep during the nap period.
To improve clarity, we have now added the following to the Discussion (page 11):

Changed from…	Changed to…
	"We note that although the combination of awake and sleep group differences in this study and previous observations from studies without any delay strongly suggest a beneficial effect of sleep rather than delay, we do not present a randomized manipulation of sleep, awake rest, and no rest."

Comment 3: p. 8 Could you elaborate on your nested models comparisons? In my understanding, a huge advantage of this approach is that you can actually statistically compare whether models are equivalent. Is a model with only sleep stage significantly worse than the full model with sleep stage and spectral slope? Is a model with only spectral slope significantly worse than the full model? From the way I read this paper, Fig. 3 displays nominal differences in AIC for individual electrodes? While this is informative, running statistical model comparisons would further strengthen these conclusions.

Response:
This is a good point. We decided to only use AIC scores for model comparison since they already provide a statistical evaluation. Although the comparison of AIC scores does not follow the Null Hypothesis Significance Testing framework, it does allow one to make a statistically meaningful conclusion about the quality of competing models, or to put it more precisely, the relative likelihoods of competing models. Specifically, we report that the best fitting model to predict insights included only the spectral slope and had an AIC score of 78.8, while the more complex model with sleep stage and slope had an AIC of 81.2. This AIC difference can be translated into a relative likelihood of the two models, and means that it is only 0.247 times as probable that the complex model is better. In other words: the slope only model is 4.06 times more likely, given the data.
In addition to these metrics, we are happy to provide results of likelihood ratio tests for all findings. This shows that adding the slope parameter to the baseline model (Insight ~ 1 + sleep stage vs. Insight ~ 1 + sleep stage + slope) has a marginal effect on modelfit (for channel F4: X^2 (1) = 3.56, p= 0.06). We also note that we do not find evidence for an improved fit when sleep stage is included in a model that already contains the slope (Insight ~ 1 + slope vs. Insight ~ 1 + sleep stage + slope; for channel F4: X^2 (2) = 1.25, p= 0.54).
We now report these additional statistics in the results section:

Changed from…	Changed to…
"Given the substantial association between sleep stages and spectral slope, we used a nested model comparison approach and tested a baseline model containing only sleep stage as a predictor for insight against a model containing sleep stage and spectral slope. This showed that spectral slope over fronto-central areas improved insight prediction compared to the baseline model (e.g., channel C4: AIC: 82.5 vs. 81.2, Fig.3B), with a steeper spectral slope relating to a higher insight likelihood (e.g., channel C4: $\beta = 1.86$). Interestingly, comparing this full model (with both sleep stage and spectral slope as predictors) with the more parsimonious model containing only the spectral slope showed that the spectral slope alone is the best predictor for insight, yielding the best of all considered models (e.g., channel C4: AIC: 81.2 vs. 78.8, Fig.3B). "	"Given the substantial association between sleep stages and spectral slope, we used a nested model comparison approach and tested a baseline model containing only sleep stage as a predictor for insight against a full model containing sleep stage and spectral slope. While the sleep stage only model performed better than a intercept only null model (for channel F4: AIC null model: 84.4, AIC sleep stage model: 82.5; likelihood ratio test between null and sleep stage model: X2(2) = 5.85, $p = 0.05$, for sleep stageN1vsWake, $\beta = 0.34$, $p = 0.60$, for sleep stageN2vsWake, $\beta = 1.57$, $p = 0.03$), we also found that spectral slope over fronto-central areas further improved insight prediction compared to the sleep stage only model for channel F4: AIC: 82.5 vs. 81.0, Fig.3B,likelihood ratio test between X2(1) = 3.56, $p = 0.06$), with a steeper spectral slope relating to a higher insight likelihood (e.g., channel F4: $\beta = -1.98$, $p = 0.07$). Interestingly, comparing this full model (with both sleep stage and spectral slope as predictors) with the more parsimonious model containing only the spectral slope showed that the spectral slope alone is the best predictor for insight, yielding the best of all considered models (e.g., channel F4: AIC: 81.0 vs. 78.2, Fig.3B for slope, $\beta = -2.53$, $p = 0.01$, likelihood ratio test that the more complex model is better: X2(2) = 1.25, $p = 0.54$."

Note that we have changed from focussing on C3 to a focus on F4, but now also provide a full table of results for frontal, central and parietal/occipital electrodes, which shows that AICs and other fit metrics of the slope-only model are consistently lower than the sleep+stage+slope model. The table is reprinted below. It appears in the SI and is referenced in the manuscript on page 10.

Channels	Models. Insight ~ ...	
	1 + sleep stage + slope	1 + slope
**Fz.**		
model fit($X^2(df)$, p, R^2)	7.95(3), 0.05, 0.02	6.58(1), 0.01, 0.06
slope parameter(β, p)	-1.70, 0.15	-2.39, 0.02
AIC	82.44	79.81
**F3.**		
model fit($X^2(df)$, p, R^2)	6.23(3), 0.10, 0.003	3.11(1), 0.08, 0.01
slope parameter(β, p)	-0.54, 0.54	-1.28, 0.09
AIC	84.16	83.28
**F4.**		
model fit($X^2(df)$, p, R^2)	9.41(3), 0.02, 0.04	8.16(1), 0.004, 0.07
slope parameter(β, p)	-1.98, 0.07	-2.53, 0.01
AIC	80.98	78.23
**Cz.**		
model fit($X^2(df)$, p, R^2)	8.41(3), 0.04, 0.03	7.03(1), 0.008, 0.06
slope parameter(β, p)	-2.00, 0.12	-2.66, 0.02
AIC	81.98	79.36
**C3.**		
model fit($X^2(df)$, p, R^2)	7.32(3), 0.06, 0.04	5.33(1), 0.02, 0.03
slope parameter(β, p)	-1.27, 0.23	-1.99, 0.03
AIC	83.07	81.06
**C4.**		
model fit($X^2(df)$, p, R^2)	8.33(3), 0.04, 0.03	6.52(1), 0.01, 0.05
slope parameter(β, p)	-1.56, 0.13	-2.12, 0.02
AIC	82.06	79.87
**Pz.**		
model fit($X^2(df)$, p, R^2)	7.58(3), 0.06, 0.02	5.82(1), 0.02, 0.05
slope parameter(β, p)	-1.57, 0.20	-2.33, 0.02
AIC	82.80	80.57
**Oz.**		
model fit($X^2(df)$, p, R^2)	6.00(3), 0.11, 0.00	2.32(1), 0.13, 0.004
slope parameter(β, p)	-0.29, 0.70	-1.00, 0.13
AIC	84.39	84.08

Table 2: Information about the full model and the slope model across frontal (Fz, F3, F4), central (Cz, C3, C4), parietal (Pz) and occipital (Oz) areas. Information include model comparison against a null model (X^2, p) and $McFaddensR^2$ adjusted for model complexity as well as the slope parameter and AIC scores.

Comment 4: p.10: please qualify that the spectral slope may offer a more fine-grained measure on some aspects related to sleep depth - I believe it is still unclear in how far it may be a "better" measure, more generally, for determining how deeply a person is asleep.

Response: We agree that there are shortcomings in explaining why the spectral slope may offer a more fine-grained measure of sleep depth. In our opinion the main benefits of the slope arise from , (a) its continuous scale and (b), due to the slope becoming steeper from N1 to N2 to N3. We made this more explicit in the changed text passage quoted below:

Changed from…	Changed to…
“The 1/f slope has been linked to consciousness and sleep depth, where a steeper slope signifies less consciousness under anaesthesia, or deeper sleep (Miskovic, MacDonald, Rhodes, & Cote, 2019; Colombo et al., 2019; Lendner et al., 2020; G. Horvath et al., 2022; Schneider et al., 2022). Compared to ordinal sleep staging, the 1/f slope is a continuous measurement that offers a more fine	“The 1/f slope has been linked to consciousness and sleep depth, where a steeper slope signifies less consciousness under anaesthesia. From wakefulness, to N1, N2 and N3 sleep, i.e., when sleep becomes deeper, the spectral slope becomes steeper (Miskovic, MacDonald, Rhodes, & Cote, 2019; Colombo et al., 2019; Lendner et al., 2020; G. Horvath et al., 2022; Schneider et al.,

grained measure of sleep depth. Hence, the fact that the spectral slope predicts insight beyond sleep stages alone supports the idea that deeper sleep is needed for insight."	2022). Since the 1/f slope is a continuous measurement and tracks different brain states during sleep on a short timescale (Lendner et al., 2024), it potentially offers a more fine grained measure of sleep depth. Hence, the fact that the spectral slope predicts insight beyond sleep stages alone suggests that deeper sleep is needed for insight. "

Comment 5: Discussion: Please elaborate on how renormalizing excitability may translate to spectral slope and the current findings. The steep spectral slopes during sleep might reflect an active inhibition process that then leads to renormalization of excitability. The idea of synaptic homeostasis would suggest a flatter E/I slope after sleep (i.e. renormalization) than before sleep (flatter slope = higher excitation, steeper slope higher inhibition), if I reason correctly? This is a complex matter and it may be helpful for readers unfamiliar with these concepts to elaborate on the assumed mechanisms and how they translate to inhibition/excitation in neural networks.

Response:
We thank the reviewer for critical reading of the manuscript and addressing this important point regarding the relationship of regularisation and synaptic renormalisation.
We have now added the following paragraphs to the manuscript in order to enhance clarity regarding this topic.

In the Discussion on page 11:

Changed from…	Changed to…
" Our previous computational work (Löwe et al., 2024) pointed towards a role of regularisation and noise for the formation of insight. Proponents of the synaptic homeostasis hypothesis (Tononi & Cirelli, 2003, 2006, 2014) have related regularisation to synaptic downscaling (Hoel, 2021), a process that regulates synaptic strength depending on the synapses' firing rates during wake. By pruning synaptic connections with low activity, overall excitability is renormalised during sleep (Turrigiano & Nelson, 2004; Olcese, Esser, & Tononi, 2010; Hashmi, Nere, & Tononi, 2013). Computational work	"Regularisation is a model simplification process that is commonly used in machine learning to improve performance and avoid overfitting (Liu et al., 2020). In the context of neural networks, regularisation shrinks or eliminates in particular weak weights between neurons. On a molecular level, this renormalisation has been linked to synaptic downscaling during sleep (Gao et al., 2017), a process that maintains a synaptic firing homeostasis by adjusting synaptic weights based on activity (De Vivo et al., 2017). It has been specifically argued that by regulating synaptic strength depending on the neurons' firing rates during wake, this

correlated this excitation-inhibition (E/I) balance with the spectral slope of aperiodic EEG activity (Gao, Peterson, & Voytek, 2017). Beyond just being a fine grained measure of sleep depth, the 1/f slope might thus reflect regularisation, which potentially plays an important role in generating insight”	scaling process can aid stable energy requirements and may avoid memory interference (De Vivo et al., 2017). Proponents of the synaptic homeostasis hypothesis (Tononi & Cirelli, 2003, 2006, 2014) have linked regularisation to synaptic downscaling (Hoel, 2021), since both processes reduce weights. The selective, activity-dependent renormalisation of synapses through regularisation during sleep might thus be an ideal candidate for gist extraction of relevant information and therefore contribute to insight-like learning phenomena.”

Comment 6: Discussion: following up on the authors' thoughts on proposed mechanisms, particularly renormalization, I agree that insight might be facilitated in the N2 participants because they face the task with a "cleaner slate" after the nap. This is a different mechanism than that suggested by the original sleep and insight study, linking reactivation with gaining insight over sleep. It does fit suggestions in the insight literature that e.g. a relaxation of previously established constraints might underlie at least part of the processes leading to insight. I would really appreciate if the authors could discuss this in more detail. It addresses a new angle in sleeps role in insight and may thus encourage relevant discussion in the field.

Response:
We thank the reviewer for raising this important point.
To elaborate on the potential sleep mechanisms fostering insight, we have added the following to the Discussion on page 13:

Changed from…	Changed to…
	“It is important to note that regularisation only reflects one of several possible mechanisms that can foster insights and are not mutually exclusive. As discussed above, regularisation can lead to a “cleaner slate” after a nap, akin to a process that relaxes previously established constraints, and can lead to gist extraction. Another mechanism that could foster insight is replay, the reactivation of sequential experience during sleep or wake (Foster, 2017), which can be used to recombine previously separate experiences and thereby promote novel inferences (Schwartenbeck et al., 2023; Olafsdottir et al., 2015).

	Replay on the other hand, might be beneficial for generating new connections, such as would be required for insight puzzles. It would also be expected that based on this mechanism, subjects should know the solution upon waking and restarting the task.”

Comment 7: Supplemental Information: It is great that the reported results hold when considering participants' classification on how deeply they slept and an automatic sleep stage classifiers categorization, but it is unclear to me, what these analyses add to the results reported in the main text. Manual sleep scoring still is the gold standard in the field and either algorithm based scoring or subjective ratings should mainly add noise to the data? Maybe I am missing an obvious point here, if yes, please consider adding a more explicit reasoning on why these additional analyses were run and what further information they provide to the paper.

Response:
We should have motivated the reason for different scoring techniques more strongly. Algorithm-based scoring or subjective ratings add more noise to the data but just if manual sleep scoring is considered as the truth/more correct method. Even though manual sleep scoring is still the golden standard in the field, it is exclusively based on a subjective classification and thus, cannot necessarily be considered as the truth/more correct method. To rule out the possibility of the scoring technique driving our findings, we applied different scoring techniques and show consistency of our findings across them. Another benefit of algorithm-based scoring is that it is per design fully blind to any other results.

We made the following changes to the manuscript, where we describe the motivation for the different scoring techniques on page 4:

Changed from…	Changed to…
“We validated the manual sleep stage scoring with a convolutional neural network trained on external polysomnography data (U-Sleep, Perslev et al. 2021). This categorisation correlated highly with manual scoring, $r(66) = 0.82$, $p < 0.001$), and results reported here can be replicated qualitatively using this alternative approach (see Supplemental Information (SI)). Similarly, splitting participants based on subjective sleep reports also results in the same pattern of results (see Fig 5, SI), although subjective reports did not match objective sleep staging closely (see SI).”	“Since manual sleep stage scoring depends on subjective classification, we validated the manual sleep stage scoring with a convolutional neural network trained on external polysomnography data (U-Sleep, Perslev et al. 2021). This categorisation correlated highly with manual scoring, $r(66) = 0.82$, $p < 0.001$), and results reported here can be replicated qualitatively using this alternative approach (see Supplemental Information (SI)). To further argue for the reported results being independent of the sleep stage scoring technique, we split participants based on their subjective sleep

	reports. Again, the reported results can be replicated qualitatively (see Fig 5, SI), although subjective reports did not match objective sleep staging closely (see SI).”

Comment 8: Supplemental Info/Methods: maybe I missed it, but please report that there were no differences in vigilance (PVT) across groups in a brief statement in the main text.

Response:
We thank the reviewer for raising this important point.
We have now added the results of the PVT to the results section on page 6.

Changed from…	Changed to…
“No effects on the corresponding reaction times could be found (N2 vs. Wake: M = 757.6 ± 48ms vs M = 809 ± 35ms, t(12.8) = -0.86, p = 0.4, d = 0.43, N2 vs. N1: M = 757.6 ± 48ms vs M = 787.8 ± 45ms, t(13.9) = -0.46, p = 0.66, d = 0.23, Fig4B.”	“No effects on the corresponding reaction times could be found (N2 vs. Wake: M = 757.6 ± 48ms vs M = 809 ± 35ms, t(12.8) = -0.86, p = 0.4, d = 0.43, N2 vs. N1: M = 757.6 ± 48ms vs M = 787.8 ± 45ms, t(13.9) = -0.46, p = 0.66, d = 0.23, Fig4B. Finally, there was no significant difference in the vigilance between Wake, N1 and N2 (assessed via reaction times in a psychomotor vigilance task, PVT, before and after the nap. linear model: rtlog ~ 1 + sleep stage + time point; for all beta: -0.045 < beta < 0.031, all p >0.2; Details see Methods and Fig8,SI)”

Comment 9: I am not a native English speaker but I stumbled across the following phrases and wanted to draw the authors' notice to them:
- On a behavioral level At a behavioral level, p.1
- Similarly to the NRT, Similar to the NRT p.2
- "Having an insight"? Experiencing insight? Moment of insight? P.1, p.3

Response:
We thank the reviewer for careful reading of the manuscript. After consultation with a native speaker, we have kept the expressions as they were.

Reviewer 2

Comment 1: As I see, the concept of "regularization" is of central importance as it provides the rationale for analyzing aperiodic EEG activity. It should be more clearly described and defined such that readers (lacking computational expertise) understand how it relates to neuronal network activity.

Response:
We thank the reviewer for raising this important point, which was also brought up by Reviewer 1 (see R1's Comments 5 and 6)
We have now introduced and motivated the concept of regularisation in more depth in the introduction (page 2), and offer a more detailed discussion about distinctions to other concepts and implications.

In the Introduction on page 2 we write:

Changed from…	Changed to…
	"Regularisation is a technique commonly used in machine learning that involves shrinking weights towards zero, in an attempt to avoid overfitting (Liu et al., 2020). Our interest in regularisation is based on the observation that homeostatic plasticity processes during sleep can lead to global shrinkage of synaptic strengths (De Vivo et al., 2017), in a manner that is broadly reminiscent of regularisation (Hoel, 2021). This shrinkage could be critical for insights in several ways. Our own modelling work has shown that regularisation causes an initial suppression of irrelevant features that leads to the delayed and abrupt nature of knowledge development during insight (Löwe et al., 2024). Additionally, we speculate that a global downscaling of synaptic weights during sleep could lead to a "cleaner slate" that might facilitate insight post nap."

Comment 2: I did not understand (in the Discussion) how synaptic renormalization leads to increased regularization. Moreover, synaptic renormalization is commonly ascribed to SWS (e.g., Liu, Niethard et al. 2024, PLoSBiol). I would expect insight to be related to SWA-related parameters If synaptic renormalization is the critical factor. Overall, the discussion regard this point is not convincing and needs to be sharpened.

The essential feature of renormalisation is that any net increases in neuronal firing that resulted from encoding awake experiences are offset by a homeostatic process that adjusts synaptic strengths in a way that preserves average firing rates and avoids runaway excitation. To the extent that learning results in LTP and therefore more excitatory

connections (Bliss & Collingridge, 1993), renormalisation is induced by synaptic downscaling (Tononi & Cirelli, 2003, 2006, 2014), which, like regularisation, reduces weights / synaptic strengths, thereby aiding generalisation and stable energy requirements. Although this link between renormalisation and regularisation is evident, we agree that many aspects are unclear and warrant further study. We have adapted the introduction and discussion to make our argument more clear and also communicate the knowledge gaps (see below).

In the Discussion on page 11:

Changed from…	Changed to…
	"Regularisation is a model simplification process that is commonly used in machine learning to improve performance and avoid overfitting (Liu et al., 2020). In the context of neural networks, regularisation shrinks or eliminates in particular weak weights between neurons. On a molecular level, this renormalisation has been linked to synaptic downscaling during sleep (Gao et al., 2017), a process that maintains a synaptic firing homeostasis by adjusting synaptic weights based on activity (De Vivo et al., 2017). It has been specifically argued that by regulating synaptic strength depending on the neurons' firing rates during wake, this scaling process can aid stable energy requirements and may avoid memory interference (De Vivo et al., 2017). Proponents of the synaptic homeostasis hypothesis (Tononi & Cirelli, 2003, 2006, 2014) have linked regularisation to synaptic downscaling (Hoel, 2021), since both processes reduce weights and support generalisation. The selective, activity-dependent renormalisation of synapses through regularisation during sleep might thus be an ideal candidate for gist extraction of relevant information and therefore contribute to insight-like learning phenomena."

We agree with the reviewer that the link between renormalisation and SWS warrants deeper discussion and have made the following changes in the Discussion (page 12):

Changed from…	Changed to…
"It should be noted, however, that to date it is unclear if synaptic downscaling occurs during NREM sleep. Some evidence has linked E/I balance adjustments to REM sleep Lendner et al., 2023), while evidence for synaptic downscaling during NREM sleep has remained indirect (Suppermpool et al., 2024, Norimoto et al., 2018)."	"It should be noted, however, that to date it is unclear if synaptic downscaling occurs during NREM sleep. Some evidence has linked E/I balance adjustments to REM sleep Lendner et al., 2023), but also to slow-wave sleep (Liu et al., 2024), while further evidence for synaptic downscaling during NREM sleep has remained indirect

	(Suppermpool et al., 2024, Norimoto et al., 2018).”<br>“While our nap intervention was not sufficiently long for participants to reach N3 sleep, our results are in line with several findings suggesting that deeper sleep more generally plays a special role in the origins of insight. Increased alpha band activity during slow wave sleep (SWS), for instance, has been found to be predictive of post sleep insight, potentially reflecting representational restructuring happening during deeper sleep (Yordanova et al., 2012). Another study found a SWS-specific effect in the beta frequency range, but interpreted particularly the oscillatory 10 Hz patterns during SWS to imply neocortical read-out of implicitly learned information stored in the hippocampus (Verleger et al., 2013). While we do not directly observe SWS in our study, and individual frequency bands such as alpha did not predict insight, the evidence seems to broadly suggest that deeper forms of sleep yield bigger benefits for insights. How this can be reconciled with findings from Lacaux and others has yet to be determined.”

Comment 3: Was the aperiodic slope calculated for the whole recording of an individual? Or was it for the N2 group only calculated for epochs with N2 and the N1 group only for epochs with N1 and so on? This issue is also related to the second question of how much N1 sleep did the N1 group have? For the N2 group the distribution of sleep stages should be reported in more detail. For example, it might also be interesting to know if some participants reached N3.

Response:
We agree that these questions should be explicitly answered in the manuscript: First, we made it more clear that we analysed the aperiodic slope across the whole period, independently of the sleep stage. We decided to do it this way since we wanted to statistically test for a contribution of the aperiodic slope on insight independently of sleep stages. Or in other words, without any information about sleep stages, how predictive is the aperiodic slope for insight.
Second, we also added information about sleep characteristics for all groups in the supplemental information.

Please note that we changed the length of the power spectra from 4 to 6 seconds since we realised that we made a mistake in reporting the length.

<table>
<tr><td>Changed from…</td><td>Changed to…</td></tr>
<tr><td>Results</td><td></td></tr>
<tr><td>“We quantified aperiodic neural activity by the spectral slope of the power spectrum in log-log space (FOOOF algorithm by Donoghue et al., 2020, range 1-45Hz, 0.2Hz frequency resolution, 4sec epochs with 50% overlap).”</td><td>“We quantified aperiodic neural activity during the entire 20 min nap period by the spectral slope of the power spectrum in log-log space (FOOOF algorithm by Donoghue et al., 2020, range 1-45Hz, 0.2Hz frequency resolution, 6sec epochs with 50% overlap).”</td></tr>
<tr><td>Methods</td><td></td></tr>
<tr><td>“To obtain estimates of aperiodic activity, the spectral slope parameter x (reflecting the slope of the power spectrum) was used. Data were segmented into 4 second epochs with an overlap of 50%.”</td><td>“To obtain estimates of aperiodic activity, the spectral slope parameter x (reflecting the slope of the power spectrum) was used. Data of the whole 20 minute nap period were segmented into 6 second epochs with an overlap of 50%.”</td></tr>
<tr><td>Results</td><td></td></tr>
<tr><td>“This analysis showed that during the 20-minute nap period 28 participants reached N2 sleep, 22 reached only N1 sleep, and 18 subjects remained awake.”</td><td>“This analysis showed that during the 20 minute nap period 28 participants reached N2 sleep, 22 reached only N1 sleep, and 18 subjects remained awake (Please note that one subject spent 1 min in N3 sleep, but was nevertheless included in the N2 group; for sleep characteristics see Supplemental Information (SI).”</td></tr>
<tr><td>Supplemental Information</td><td></td></tr>
<tr><td></td><td>Supplemental Information
<table>
<tr><td>Sleep stage (in min)</td><td>Groups</td><td></td><td></td></tr>
<tr><td></td><td>Wake group</td><td>N1 group</td><td>N2 group</td></tr>
<tr><td>Wake</td><td>20</td><td>17.29±0.67
(7.5-19.5)</td><td>9.48±0.85
(1.5-17.5)</td></tr>
<tr><td>N1</td><td></td><td>2.70±0.67
(0.5-12.5)</td><td>4.32±0.42
(1-9)</td></tr>
<tr><td>N2</td><td></td><td></td><td>6.20±0.75
(1-15)</td></tr>
</table>
Table 1: The averaged duration (in minutes±SEM; minimum and maximum as range in round brackets below) for each group (Wake, N1 and N2 group) spent in each sleep stage (Wake, N1 and N2). Please note that one participant spent 1 min in N3 sleep, but was nevertheless included in the N2 group. N3 duration is not shown here.</td></tr>
</table>

Comment 4: The authors provide bayes factors to strengthen their results. Please provide some information on this analysis. Was it done in R, which library was used? Where the default priors used?

Response:
We thank the reviewer for raising this point.
Bayes Factors were analysed in R using the BFpack library using fitted GLMs for the respective hypotheses as inputs. Instead of the default priors, the respective hypotheses were included as constraints.

We now clarify this in the Methods section on page 18:

Changed from…	Changed to…
	"Bayes Factors were analysed in R using the BFpack library using fitted GLMs for the respective hypotheses as inputs. The respective hypotheses were included as constraints."

Comment 5: For the GLMs I was wondering what kind of contrasts were used, especially for factors with 3 levels? Were these contrasts considered at all, or just the post-hoc comparisons? For the GLMs more information on the model output is desirable. Of course, AIC is helpful for model comparison, however, especially for the models reported in Fig. 3b, one just knows that one model is a better fit than the other but the information whether the better fit actually is predictive is missing. I understand that more specific information is just reported for one exemplary channel, but please provide more information than just the beta coefficient.

Response:
We agree that adding this information is helpful. For more information about the parameters and the GLMs, we adapted the results and method section by report Mc Faddens R^2 for the full model (with sleep stage + slope as predictors) as well as for the slope model (just containing the slope as a predictor, R2 adjusted for model complexity). In addition, we added a table to the supplement where we list statistical information about selected channels and to which we now refer in the results. Further, we adjusted Figure 3b to highlight only channels for which the difference in AIC scores is < 0 *and* where the better fit significantly predicts the data better than the null model (goodness of fit test vs. null model, $p < .05$). Please note that due to an indexing error the p-values in Figure 3 A and D slightly changed. In the manuscript, we report statistics for channel F4.

Changed from…	Changed to…
Results	
“Given the substantial association between sleep stages and spectral slope, we used a nested model comparison approach and tested a baseline model containing only sleep stage as a predictor for insight against a model containing sleep stage and spectral slope. This showed that spectral slope over fronto-central areas improved insight prediction compared to the baseline model (e.g., channel C4: AIC: 82.5 vs. 81.2, Fig.3B), with a steeper spectral slope relating to a higher insight likelihood (e.g., channel C4: $\beta = 1.86$).”	“Given the substantial association between sleep stages and spectral slope, we used a nested model comparison approach and tested a baseline model containing only sleep stage as a predictor for insight against a full model containing sleep stage and spectral slope. While the sleep stage only model performed better than an intercept only null model (for channel F4: AIC null model: 84.4, AIC sleep stage model: 82.5; likelihood ratio test between null and sleep stage model: X2(2) = 5.85, $p= 0.05$, for sleep stageN1vsWake, $\beta = 0.34$, $p= 0.60$, for sleep stageN2vsWake, $\beta = 1.57$, $p= 0.03$), we also found that spectral slope over fronto-central areas further improved insight prediction compared to the sleep stage only model for channel F4: AIC: 82.5 vs. 81.0, Fig.3B,likelihood ratio test between X2(1) = 3.56, $p= 0.06$), with a steeper spectral slope relating to a higher insight likelihood (e.g., channel F4: $\beta=-1.98$,$p= 0.07$).”
Methods	
“AIC scores were used to assess the best model fit.”	“AIC scores and likelihood ratio tests were used to assess the best model fit in a hierarchical manner, while adjusted McFadden R2 scored are reported to indicate total model fit.”
Results	
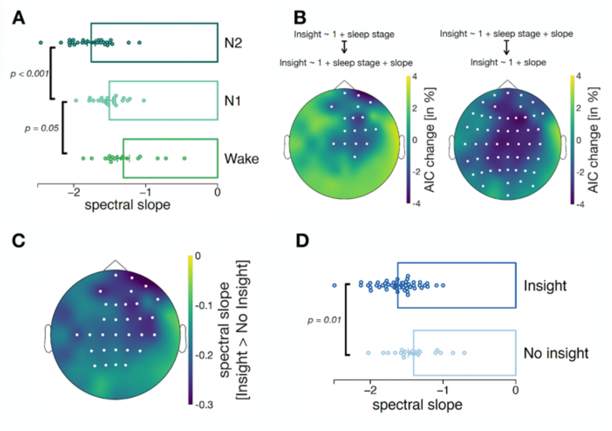<br>Figure 3. A: The spectral slope significantly decreased from Wake to N1 to N2, as	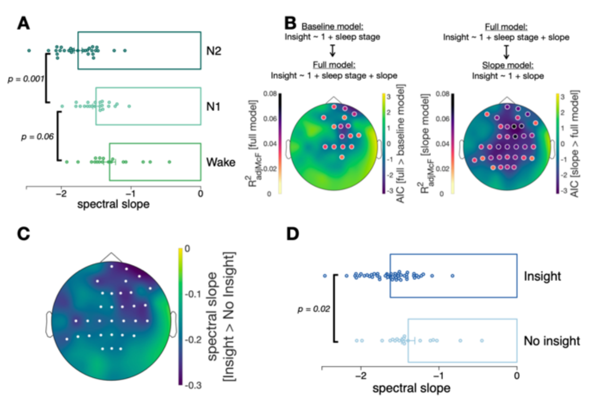<br>Figure 3. A: The spectral slope significantly decreased from Wake to N1 to N2, as expected. For the

expected. For the corresponding topoplot see Supplemental Information, Fig.6. B: Topographies of model comparison results testing a model of interest that included sleep stage and spectral slope (left) or only spectral slope (right) against a baseline model (left: insight ~ 1 + sleep stage, right: insight ~ 1 + sleep stage + slope). Shown are channel-wise model fit improvements obtained by including the spectral slope (left) or removing sleep stage (right; AIC in percentage, negative numbers indicate better fit of the main models). Channels with AIC differences < 0 were located over fronto-central areas (left) or central areas (right) and are highlighted in white. C: The spectral slope was significantly steeper (i.e., more negative) for participants with insight vs. participants without insight, over fronto-central areas. All channels that are part of the significant cluster are highlighted in white. D: The comparison of the spectral slope between participants with vs. without an insight for channel C4 (part of the significant cluster in C).	corresponding topoplot see Supplemental Information, Fig.6. B: Topographies of model comparison results testing the full model, including sleep stage and spectral slope, vs. a baseline model, including just sleep stage (left) and the slope model, including just the slope, vs. the full model, including sleep stage and spectral slope (right). Shown are channel-wise model fit improvements (see colour scale on the right side) obtained by including the spectral slope (left, AIC differences with negative values indicate a better fit of the full model) or removing sleep stage (right, AIC differences with negative values indicate a better fit of the slope model). For channels with AIC differences < 0 and a significant model prediction of the full (left) or slope model (right), Mc Faddens R^2 adjusted for model complexity is shown as a colour-coded dot (see colour legend left) . See Supplemental Information for model information of frontal, central, parietal and occipital channels. C: The spectral slope was significantly steeper (i.e., more negative) for participants with insight vs. participants without insight, over fronto-central areas. All channels that are part of the significant cluster are highlighted in white. D: The comparison of the spectral slope between participants with vs. without an insight for channel C4 (part of the significant cluster in C)
Supplemental Information	

Channels	Models. Insight ~ ...	
	1 + sleep stage + slope	1 + slope
**Fz.**		
model fit($X^2(df)$, p, R^2)	7.95(3), 0.05, 0.02	6.58(1), 0.01, 0.06
slope parameter(β, p)	-1.70, 0.15	-2.39, 0.02
AIC	82.44	79.81
**F3.**		
model fit($X^2(df)$, p, R^2)	6.23(3), 0.10, 0.003	3.11(1), 0.08, 0.01
slope parameter(β, p)	-0.54, 0.54	-1.28, 0.09
AIC	84.16	83.28
**F4.**		
model fit($X^2(df)$, p, R^2)	9.41(3), 0.02, 0.04	8.16(1), 0.004, 0.07
slope parameter(β, p)	-1.98, 0.07	-2.53, 0.01
AIC	80.98	78.23
**Cz.**		
model fit($X^2(df)$, p, R^2)	8.41(3), 0.04, 0.03	7.03(1), 0.008, 0.06
slope parameter(β, p)	-2.00, 0.12	-2.66, 0.02
AIC	81.98	79.36
**C3.**		
model fit($X^2(df)$, p, R^2)	7.32(3), 0.06, 0.04	5.33(1), 0.02, 0.03
slope parameter(β, p)	-1.27, 0.23	-1.99, 0.03
AIC	83.07	81.06
**C4.**		
model fit($X^2(df)$, p, R^2)	8.33(3), 0.04, 0.03	6.52(1), 0.01, 0.05
slope parameter(β, p)	-1.56, 0.13	-2.12, 0.02
AIC	82.06	79.87
**Pz.**		
model fit($X^2(df)$, p, R^2)	7.58(3), 0.06, 0.02	5.82(1), 0.02, 0.05
slope parameter(β, p)	-1.57, 0.20	-2.33, 0.02
AIC	82.80	80.57
**Oz.**		
model fit($X^2(df)$, p, R^2)	6.00(3), 0.11, 0.00	2.32(1), 0.13, 0.004
slope parameter(β, p)	-0.29, 0.70	-1.00, 0.13
AIC	84.39	84.08

Table 2: Information about the full model and the slope model across frontal (Fz, F3, F4), central (Cz, C3, C4), parietal (Pz) and occipital (Oz) areas. Information include model comparison against a null model (X^2, p) and $McFaddensR^2$ adjusted for model complexity as well as the slope parameter and AIC scores.

Comment 6: What was the rationale for modelling sleep stage and spectral slope additively. Did the authors check whether there are any interactions? Wouldn't it be possible that only in the N2 group the slope is predictive of insight?

Response:
This is a very valid question and we should have made this more explicit in the manuscript. Indeed, we consciously decided to not include the interaction term between sleep stage and spectral slope since the spectral slope was calculated across all sleep stages. For example, if one participant was classified as N2, the spectral slope of this participant was still calculated across wake, N1 and N2. Consequently, a clear interpretation of the interaction term in this case is not possible.

Comment 7: Please provide more information regarding the settings for the analysis using FOOOF. The frequency range for the power calculation is not necessarily the same as for the FOOOF calculation. Was a "knee" fitted? What was the maximum number of peaks to be fitted, the maximum peak height etc.

Response: We agree that more information could be provided. We adapted the method section and included the information.

Changed from…	Changed to…
“where b is the y intercept, k is the knee	“where b is the y intercept, k is the knee parameter and x is the slope parameter. The knee parameter was set to 0 since we did

parameter and x is the slope parameter. ”	not expect the aperiodic activity to change across the frequency range. Lower frequencies (< 1 Hz) often show a plateau in the power spectrum resulting in the need to fit a knee (Gerster et al. 2022). To avoid this, the lower limit of the frequency range was set to 1 Hz. All other settings for the fit (including the peaks for the periodic component) were kept at their default values (maximum number of peaks = 3, minimum height of a peak = 3 dB, limits of peak width = 0.5-12 Hz).”

Comment 8: Analyses of aperiodic corrected power: since the authors preregistered to explore an association between delta power and insight likelihood, I was wondering why this analysis is missing in the manuscript.

Response:
We thank the reviewer for carefully reading the preregistration and raising this important point.
We indeed contrasted participants with and without insight across all frequencies, including delta, and did not observe any differences. In addition, we extracted power in the delta frequency range (1-4Hz) and found that it did not explain insight beyond sleep stages (AIC for model containing only sleep stages = 82.5; AIC for model with sleep stages + delta power at channel C4 = 84.4). This is reported in SI and Supplement Figure 7D and we now made this more explicit in the Supplementary Material on page 24 and 27 (see below) and also mention it briefly in the main manuscript.
While it is essential to report all results from pre-registered analyses, we did not expand on this result since we found no evidence for a role of delta when comparing Wake, N1 and N2 sleep in a data-driven fashion (across all frequency ranges including alpha and delta, 1-20Hz). However, we agree that we should include the pre-registered analysis in the supplement as well. Together, made the following changes and additions:

Changed from…	Changed to…
“A complementary pattern emerges when directly contrasting participants with and without insight across the whole frequency range. No significant differences were observed (cluster-based permutation test, pcluster = 0.31).”	“A complementary pattern emerges when directly contrasting participants with and without insight across the whole frequency range. No significant differences were observed, neither in the alpha, spindle, delta or any other frequency range (cluster-based permutation test, pcluster = 0.31).”
“Post hoc cluster-based permutation tests revealed a positive and negative cluster in the alpha (5.8-11.3Hz) and sleep spindle frequency range (11.5-15.2Hz), respectively	“Post hoc cluster-based permutation tests revealed a positive and negative cluster in the alpha (5.8-11.3Hz) and sleep spindle frequency range (11.5-15.2Hz), respectively

(post-hoc cluster-based permutation test, t-statistics, Wake > N1: negative cluster, pcluster = 0.02, 10.5-14Hz; Wake > N2: positive cluster, pcluster = 0.07, 6-9Hz; negative cluster, pcluster = 0.05, 11.5-15.2Hz; N1 > N2: positive cluster, pcluster = 0.007, 5.8-12.3Hz). Neither averaged power in the alpha nor in the spindle cluster explained insight beyond sleep stages (AIC for model containing only sleep stages = 82.5; AIC for model with sleep stages + alpha power at channel C4 = 84.5; AIC for model with sleep stages + spindle power at channel C4 = 84.1, Fig.7B). In line with the spectral slope analyses, we also removed sleep stages from both models. Removing sleep stages from both models resulted in a worse model fit (AIC for model with spindle power at channel C4 = 85.9; AIC for model with spindle power at channel C4 = 86.3, Fig.7C)."	(post-hoc cluster-based permutation test, t-statistics, Wake > N1: negative cluster, pcluster = 0.02, 10.5-14Hz; Wake > N2: positive cluster, pcluster = 0.07, 6-9Hz; negative cluster, pcluster = 0.05, 11.5-15.2Hz; N1 > N2: positive cluster, pcluster = 0.007, 5.8-12.3Hz).Since we also wanted to test the a priori hypothesis of delta power predicting insight (see pre-registration), we additionally extracted power in the delta frequency range (1-4Hz). Neither averaged power in the alpha nor in the spindle cluster nor in the delta frequency range explained insight beyond sleep stages (AIC for model containing only sleep stages = 82.5; AIC for model with sleep stages + alpha power at channel C4 = 84.5; AIC for model with sleep stages + spindle power at channel C4 = 84.1, Fig7B; AIC for model with sleep stages + delta power at channel C4 = 84.4). In line with the spectral slope analyses, we also removed sleep stages from both models. Removing sleep stages from both models resulted in a worse model fit (AIC for model with spindle power at channel C4 = 85.9; AIC for model with spindle power at channel C4 = 86.3, Fig.7C; AIC for model with delta power at channel C4 = 85.6)."

Reviewer 3

Comment 1: The sleep duration seems very short and they did not provide the specific sleep parameters (e.g.: the time of wake, N1 and N2). The classification of subjects only by reaching N2 is concerning, as extremely brief periods in N2 (such as 1-2 minutes) could undermine the validity of the results.

Response:
We thank the reviewer for raising this important point and pointing out that specific sleep parameters were missing.
On average, the N2 group spent 9.5 minutes awake, 4.3 minutes in N2 and 6.2 minutes in N2 (range 1-15 minutes). In fact, only 4 out of 28 participants classified as the N2 group spent less than 2 minutes in that sleep stage. To address the reviewer's concern that extremely brief periods undermine the results, we have excluded participants with less than 2 minutes of N2 (4 participants). This shows that the adjusted N2 group still shows a larger proportion of insights compared to the N1 group (insight likelihood $N2_{adjusted}$ = 87.5% [21/24] vs. N1 = 63.6% [14/22], Fisher's exact test: p = 0.086; for completeness: insight likelihood with the whole N2 sample = 85.7% [24/28]), hence suggesting that our results are not driven by extremely brief periods of N2. We now mention this information in the main manuscript.

We also note that based on our pre-registration, we followed the same experimental design and rationale of the analyses of the original study (Lacaux et al., 2021) where brief pauses of N2 were classified as N2.
Generally speaking, not much is known about how the effects of short and long sleep periods on cognition differ. While sleep-related benefits on cognitive processes in general are well documented, most studies rely on a full night of sleep and the minimum duration of sleep for being beneficial has rarely been tested. There is evidence that very short naps (30 minutes) can show sleep-related benefits for cognition (Leong et al., 2023), although these cognitive processes can not necessarily be translated into insight. Our results are therefore particularly interesting in that they open up the question of how much sleep is actually needed to be beneficial.
Lastly, we would like to highlight that our results indicate that a more data-driven measure, i.e., the 1/f slope, is superior for predicting insight, which could indicate that the arbitrary categorisation into groups is suboptimal in general, in line with the reviewers idea.

Changed from…	Changed to…
"We thus find no evidence that N1 sleep promotes insight as reported by Lacaux et al. (2021). Instead, in our data N2 sleep showed a significant association with insight frequency. "	"We thus find no evidence that N1 sleep promotes insight as reported by Lacaux et al. (2021). Instead, in our data N2 sleep showed a significant association with insight frequency. Please note that the results do not qualitatively change when participants with very short episodes of N2 sleep (< 2min) are excluded (excluded participants = 4; insight likelihood of N2adjusted= 87.5% [21 of 24

<table>
<tr><td></td><td>participants with insight], Fisher’s exact test N2adjusted vs. N1, p= 0.09; Fisher’s exact test N2adjusted vs. W, p= 0.03).”</td></tr>
<tr><td>“This analysis showed that during the 20-minute nap period 28 participants reached N2 sleep, 22 reached only N1 sleep, and 18 subjects remained awake.”</td><td>“This analysis showed that during the 20-minute nap period 28 participants reached N2 sleep, 22 reached only N1 sleep, and 18 subjects remained awake (Please note that one subject spent 1 min in N3 sleep, but was nevertheless included in the N2 group; for sleep characteristics see Supplemental Information (SI).”</td></tr>
<tr><td></td><td>Supplemental Information
<table>
<tr><th>Sleep stage (in min)</th><th colspan="3">Groups</th></tr>
<tr><th></th><th>Wake group</th><th>N1 group</th><th>N2 group</th></tr>
<tr><td>Wake</td><td>20</td><td>17.29±0.67
(7.5-19.5)</td><td>9.48±0.85
(1.5-17.5)</td></tr>
<tr><td>N1</td><td></td><td>2.70±0.67
(0.5-12.5)</td><td>4.32±0.42
(1-9)</td></tr>
<tr><td>N2</td><td></td><td></td><td>6.20±0.75
(1-15)</td></tr>
</table>
Table 1: The averaged duration (in minutes±SEM; minimum and maximum as range in round brackets below) for each group (Wake, N1 and N2 group) spent in each sleep stage (Wake, N1 and N2). Please note that one participant spent 1 min in N3 sleep, but was nevertheless included in the N2 group. N3 duration is not shown here.</td></tr>
</table>

Comment 2: I don't know whether all the subjects did the experiment at the same time of day. Could circadian rhythms have influenced the findings?

Response:
We thank the reviewer for raising this point.
All participants were tested at the same time of day with the experiment starting at 1pm.
Consequently, the nap period started around 2.30 pm for all participants.
This had been stated in the Methods section of the manuscript already, but we now added this as the first sentence of the Behavioural Procedure for increased clarity:

Changed from…	Changed to…
“The experimental procedure consisted of 3 parts: (1) a first behavioural session of about 25 minutes, including the PVT and 400 trials of the RDK task, followed by (2) a nap of 20 minutes and (3) a second behavioural session of about 30 minutes, including the PVT and 500 more trials of the RDK task. ”	“For all participants, the experimental procedure started at the same time (1 pm) to rule out potential time of day confounds. The experimental procedure consisted of 3 parts: (1) a first behavioural session of about 25 minutes, including the PVT and 400 trials of the RDK task, followed by (2) a nap of 20 minutes and (3) a second behavioural session of about 30 minutes, including the PVT and 500 more trials of the RDK task. ”

Comment 3: It isn't clear why the current results are so different from the prior findings regarding N1 and insight. This is discussed, but I feel it deserves a more in depth treatment.

Response: We agree that this is an interesting point. However, since there are only a few studies investigating the relationship between N1 sleep and cognitive processes, it remains challenging to explain differences. This is why we also emphasise the need for replications and further (preregistered) studies specifically investigating the relationship between sleep and insight.
Yet, there is evidence that does suggest a link between deeper sleep (N2 sleep but also N3 sleep) and insight, which we mention in the Discussion (page 12). Another potential reason are task differences, which we also mention in greater detail in the Discussion (page 12).

Discussion, page 12:

Changed from…	Changed to…
	"While our nap intervention was not sufficiently long for participants to reach N3 sleep, our results are in line with several findings suggesting that deeper sleep more generally plays a special role in the origins of insight. Increased alpha band activity during slow wave sleep (SWS), for instance, has been found to be predictive of post sleep insight, potentially reflecting representational restructuring happening during deeper sleep (Yordanova et al., 2012). Another study found a SWS-specific effect in the beta frequency range, but interpreted particularly the oscillatory 10 Hz patterns during SWS to imply neocortical read-out of implicitly learned information stored in the hippocampus (Verleger et al., 2013). While we do not directly observe SWS in our study, and individual frequency bands such as alpha did not predict insight, the evidence seems to broadly suggest that deeper forms of sleep yield bigger benefits for insights. How this can be reconciled with findings from Lacaux and others has yet to be determined."

Discussion, page 12:

Changed from…	Changed to…
	"Beyond sleep stages, different cognitive tasks might benefit from sleep in different ways – and potentially explain divergent findings regarding the relationship of sleep and insight. For instance, sleep has been found to aid in

	statistical learning (Durrant, Taylor, Cairney, & Lewis, 2011) and particularly in the extraction of hidden task regularities (Lerner & Gluck, 2019).<br>Besides the associative learning of hidden structure in the PSSST and NRT, participants also need to overcome the initially learned strategy, which could block learning of the associations required for the insight (Kamin, 1969; Mackintosh, 1973). Both downscaling of redundant information in terms of weak synaptic connections, as well as global downscaling of all synaptic weights to a speculative 'clean slate' could potentially account for the post sleep insight. In favour of the clean slate hypothesis would be that subjects often take a while after waking up to discover the hidden rule (Wagner et al. 2004, Lacaux et al., 2021), i.e. they don't wake up with the insight solution."

Comment 4: I find it surprising that insight proportions differ significantly between the groups (wake, N1, and N2), yet task performance remains unaffected. After participants learned the color rule, the task should theoretically have been easy to complete with high accuracy. I expected the N2 group to outperform the other two. Could this be due to practice effects? Despite not learning the insight rule, perhaps the participants were able to perform well after repeated trials. However, in Figure 2D, there seems to be a marked performance improvement in every group from Block 0 to Block 1.

Response:
We thank the reviewer for raising this point. As the reviewer suspects, the N2 group indeed performs better than other groups in the task more generally, as shown in the plot below that is included in the supplementary material (t-test block 5-12, N2 vs. Wake: M = 85% vs M = 76%, $t(14) = 2.06$, $p = 0.06$, $d = 1.03$; N2 vs. N1: M = 85% vs M = 81%, $t(12.1) = 1.06$, $p = 0.31$, $d = 0.53$), although this is only trending.

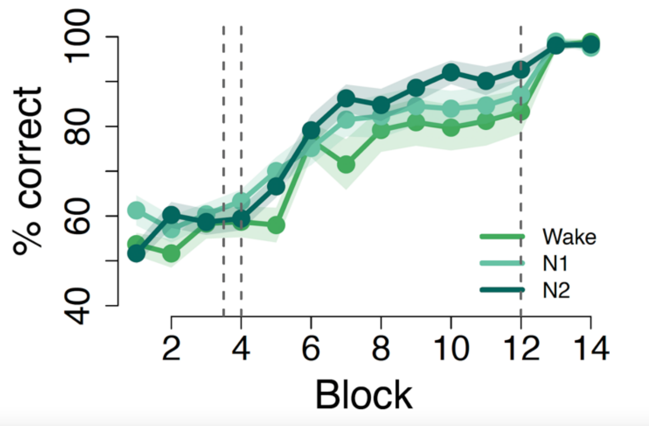

Fig 2D shows only behavior of insight subjects, which effectively adjusts for any differences in frequency of insight vs no insight subjects between the groups.

We have now clarified this in the manuscript as well by changing the title of subplot 2D and 2E to “Insight-aligned Task Performance” and “Insight-aligned Reaction Times” respectively.

Comment 5: For the FOOOF analysis, they found that the slope of 1/f, rather than the oscillation, correlates to insight likelihood. Although 1/f is normally considered as a background noise, it did have some neural information itself. However, the rationale behind why the slope alone predicts results better than a model including both slope and sleep stage was not clearly explained.

Response:Thank you for pointing out that the rationale needs clarification. Statistically speaking, the fact that the slope alone predicts insight better means that most variance of insight that is explained by the sleep stage parameter is also captured by the slope parameter. Furthermore, comparing a sleep-stage-only model with a sleep-stage-plus-slope model showed that adding the sloped increased fit; hence, the slope parameter explains additional variance above and beyond the sleep stage. Being more parsimonious and making the sleep stage parameter redundant for explaining variance of insight, the slope model is the better model.
We made the following changes to conceptually explain this:

Changed from…	Changed to…
“In conclusion, variations in aperiodic activity during a nap period predict whether participants will gain insight, with steeper spectral slopes, particularly over fronto-central areas, linked to higher insight likelihood. This association exists across sleep stages, and is stronger than previously described links between sleep stages or oscillatory power and insight. ”	“In conclusion, variations in aperiodic activity during a nap period predict whether participants will gain insight, with steeper spectral slopes, particularly over fronto-central areas, linked to higher insight likelihood. Aperiodic activity explained insight beyond sleep stages and was even sufficient to explain insight to a similar extent than aperiodic activity together with sleep stages, with the latter suggesting that aperiodic activity captures all information obtained by sleep stages. Further, the association between aperiodic activity and insight is stronger than previously described links between sleep stages or oscillatory power and insight. ”

Comment 6: A minor observation: In Fig1D, the motion coherence values on the x-axis appear inconsistent with those in Fig. 1C, e.g.: the light green one is 76% in 1c

but seem to be 45% in 1d.

Response:
We thank the reviewer for raising this point. The x-axis of Fig 1D has now been corrected.
