# Supplementary material for: N2 sleep promotes the occurrence of ‘aha’ moments in a perceptual insight task
Source: PLoS Biol. 2025 Jun 26;23(6):e3003185. doi: 10.1371/journal.pbio.3003185 (PMC12200826; doi:10.1371/journal.pbio.3003185)
Supplement: Attachment — Submitted filename: Loewe_etal_Revision2_PointbyPoint.pdf [file pbio.3003185.s003.pdf]

We thank all Reviewers for their positive feedback on our manuscript and are happy that R1 and R3 did not have any further comments.

Reviewer 2

Reviewer #2: All points raised have been satisfactorily answered. However, now knowing that the aperiodic slope has been calculated across the whole 20-min period independent of the sleep stage, I feel that the conclusions drawn in the discussion are not entirely reflected by the results. The discussion should very clearly point out again that the aperiodic slope has been calculated across a mixture of wake and sleep epochs for the N1 and N2 groups and thus reflects sleep depth only to a limited degree. Conclusions like "Hence, the fact that the spectral slope predicts insight beyond sleep stages alone suggests that deeper sleep is needed for insight." doesn't seem to be completely supported by the current analysis. I was also wondering whether the authors might add results from an analysis of the slope for the respective stages separately (i.e. considering only N1 epochs for N1 group and N2 epochs in the N2 group).

Response:
This is an important point. We agree that considering the spectral slope only for the deepest sleep stages in each group could strengthen our conclusions. We therefore have repeated all core analyses with the revised spetral slope metric (calculated based on N1 periods in the N1 group and N2 periods in the N2 group); we found strikingly similar results to our previous analyses. Below we reproduce the main Fig 3 (results when using slope of whole nap period), and the Fig. F in the SI of the revised manuscript, which uses the more specific spetral slope.

<table>
<tr>
<th>Main results when using spectral slope of entrie nap period</th>
<th>Main results when using spectral slope calculated only for deepest sleep periods</th>
</tr>
<tr>
<td>

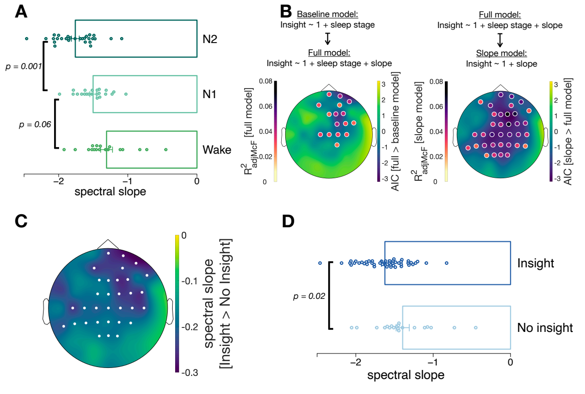

</td>
<td>

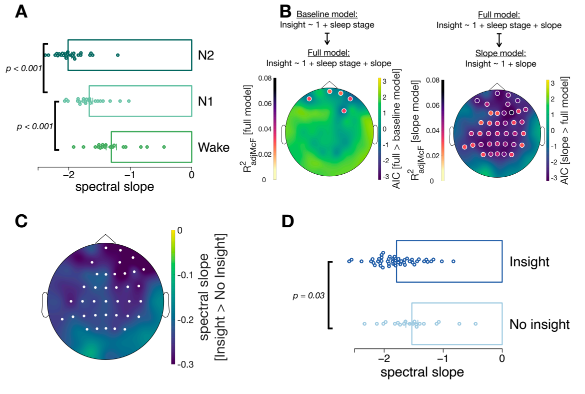

</td>
</tr>
<tr>
<td>A: The spectral slope significantly decreased from Wake to N1 to N2, as expected. For the corresponding topoplot see Supplemental Information, Fig C in S1 Text. B: Topographies of model comparison results testing the full model, including sleep stage and spectral slope, vs. a baseline model, including just sleep stage (left) and</td>
<td>Instead of calculating aperiodic neural activity during the entire 20 min nap period, here, the spectral slope was obtained from all 6sec epochs of the deepest sleep stage (e.g., N1 sleep stage for the N1 group or N2 sleep stage for the N2 group). A: The spectral slope significantly decreased from Wake to N1 to N2 (for channel C4: Wake vs. N1:M_Wake = -1.31+/-0.09</td>
</tr>
</table>

the slope model, including just the slope, vs. the full model, including sleep stage and spectral slope (right). Shown are channel-wise model fit improvements (see colour scale on the right side) obtained by including the spectral slope (left, AIC differences with negative values indicate a better fit of the full model) or removing sleep stage (right, AIC differences with negative values indicate a better fit of the slope model). For channels with AIC differences < 0 and a significant model prediction of the full (left) or slope model (right), Mc Faddens R^2 adjusted for model complexity is shown as a colour-coded dot (see colour legend left). See Supplemental Information for model information of frontal, central, parietal and occipital channels. **C**: The spectral slope was significantly steeper (i.e., more negative) for participants with insight vs. participants without insight, over fronto-central areas. All channels that are part of the significant cluster are highlighted in white. **D**: The comparison of the spectral slope between participants with vs. without an insight for channel C4 (part of the significant cluster in C). Repeating all analyses with aperiodic neural activity calculated only during the deepest sleep stage revealed comparable results, see Fig F in S1 Text.	vs. M_N1 = -1.67+/-0.06, t(30.3) = 3.52, p < 0.001, d = 1.15, N1 vs. N2: M_N1 = -1.67+/-0.06 vs. M_N2 - 2.01+/-0.05, t(46.88) = 4.40, p < 0.001, d = 1.24). **B**: Topographies of model comparison results testing the full model, including sleep stage and spectral slope, vs. a baseline model, including just sleep stage (left) and the slope model, including just the slope, vs. the full model, including sleep stage and spectral slope (right). Shown are channel wise model fit improvements (see colour scale on the right side) obtained by including the spectral slope (left, AIC differences with negative values indicate a better fit of the full model) or removing sleep stage (right, AIC differences with negative values indicate a better fit of the slope model). For channels with AIC differences < 0 and a significant model prediction of the full (left) or slope model (right), Mc Faddens R^2 adjusted for model complexity is shown as a colour-coded dot (see colour legend left). **C**: The spectral slope was significantly steeper (i.e., more negative) for participants with insight vs. participants without insight, over fronto-central areas. All channels that are part of the significant cluster are highlighted in white. **D**: The comparison of the spectral slope between participants with vs. without an insight for channel C4 (part of the significant cluster in C): Insight vs. No Insight: M_Insight = -1.79+/-0.05 vs. M_No Insight = -1.53+/-0.10, t(30.35) = -2.26, p = 0.03, d = 0.65.
